# A whole-body mathematical model of cholesterol metabolism and its age-associated dysregulation

**DOI:** 10.1186/1752-0509-6-130

**Published:** 2012-10-10

**Authors:** Mark T Mc Auley, Darren J Wilkinson, Janette JL Jones, Thomas BL Kirkwood

**Affiliations:** 1 Campus for Ageing and Vitality, Newcastle University, Henry Wellcome Biogerontology Building, Newcastle upon Tyne, NE4 5PL, United Kingdom; 2 School of Mathematics & Statistics, Newcastle University, Newcastle upon Tyne, NE1 7RU, UK; 3 Unilever R&D, Port Sunlight, Quarry Road East, Bebington, Wirral, CH63 3JW, UK

## Abstract

**Background:**

Global demographic changes have stimulated marked interest in the process of aging. There has been, and will continue to be, an unrelenting rise in the number of the oldest old ( >85 years of age). Together with an ageing population there comes an increase in the prevalence of age related disease. Of the diseases of ageing, cardiovascular disease (CVD) has by far the highest prevalence. It is regarded that a finely tuned lipid profile may help to prevent CVD as there is a long established relationship between alterations to lipid metabolism and CVD risk. In fact elevated plasma cholesterol, particularly Low Density Lipoprotein Cholesterol (LDL-C) has consistently stood out as a risk factor for having a cardiovascular event. Moreover it is widely acknowledged that LDL-C may rise with age in both sexes in a wide variety of groups. The aim of this work was to use a whole-body mathematical model to investigate why LDL-C rises with age, and to test the hypothesis that mechanistic changes to cholesterol absorption and LDL-C removal from the plasma are responsible for the rise. The whole-body mechanistic nature of the model differs from previous models of cholesterol metabolism which have either focused on intracellular cholesterol homeostasis or have concentrated on an isolated area of lipoprotein dynamics. The model integrates both current and previously published data relating to molecular biology, physiology, ageing and nutrition in an integrated fashion.

**Results:**

The model was used to test the hypothesis that alterations to the rate of cholesterol absorption and changes to the rate of removal of LDL-C from the plasma are integral to understanding why LDL-C rises with age. The model demonstrates that increasing the rate of intestinal cholesterol absorption from 50% to 80% by age 65 years can result in an increase of LDL-C by as much as 34 mg/dL in a hypothetical male subject. The model also shows that decreasing the rate of hepatic clearance of LDL-C gradually to 50% by age 65 years can result in an increase of LDL-C by as much as 116 mg/dL.

**Conclusions:**

Our model clearly demonstrates that of the two putative mechanisms that have been implicated in the dysregulation of cholesterol metabolism with age, alterations to the removal rate of plasma LDL-C has the most significant impact on cholesterol metabolism and small changes to the number of hepatic LDL receptors can result in a significant rise in LDL-C. This first whole-body systems based model of cholesterol balance could potentially be used as a tool to further improve our understanding of whole-body cholesterol metabolism and its dysregulation with age. Furthermore, given further fine tuning the model may help to investigate potential dietary and lifestyle regimes that have the potential to mitigate the effects aging has on cholesterol metabolism.

## Background

Lipid metabolism has a key role to play in human longevity and healthy ageing. This has been emphasized by recent genetic studies examining the lipoprotein phenotype in individuals with exceptional longevity [1,2]. It is highly probable such subjects with exceptional longevity and favourable lipoprotein profiles have avoided CVD. CVD is the primary cause of mortality in developed countries, with almost 40% of males and 30% of females in the United Kingdom over the age of 85 years living with the condition [3]. Of the components of lipid metabolism, elevated LDL-C has consistently stood out as a risk factor for CVD [4]. This cholesterol sub-fraction has been connected to atherosclerosis, a process regarded as the underlying pathogenesis for coronary heart disease (CHD) and stroke, the leading forms of CVD [5]. Studies have repeatedly demonstrated that regardless of physical activity levels and nutritional status, LDL-C has been shown to rise with age in both males and females in a diverse range of groups [6,7]. Understanding why LDL-C rises with age is complex; nevertheless rodent studies have indicated that increases in intestinal cholesterol absorption or possibly a decrease in the plasma clearance rate of LDL-C may have a mechanistic role to play [8-10]. In this paper we examine the hypothesis that these two mechanisms are central to understanding why LDL-C increases with age. To test this hypothesis a number of steps were required. Firstly, it was necessary to study both intestinal cholesterol absorption and LDL-C plasma clearance within an integrative framework which incorporated the other fundamental biological components of this complex system [11,12]. This required incorporating the interactions of the individual elements of this system. Secondly, it was important to investigate changes both to intestinal cholesterol absorption and LDL-C clearance over an extended period of time. Clearly it would have been difficult to test our hypothesis within this framework using conventional *in vivo* or *in vitro* techniques as such approaches can be resource intensive, expensive, time consuming, unpractical and potentially unethical [13]. Furthermore, whole-body cholesterol metabolism is a complex system with a variety of non-linear interactions among its various components, including both positive and negative feedback and complex crosstalk between elements such as cholesterol synthesis and absorption [14].

Utilizing a mechanistic mathematical model offered an alternative means of overcoming these difficulties and presented a cheap, ethical and practical way of investigating our hypothesis [13]. However, as existing computational models of cholesterol metabolism were either of an intracellular nature, lipoprotein kinetic focused, or compartmental in nature, they failed to incorporate the ageing process or address the holistic nature of our question and were thus determined to be unsuitable [15,16]. Consequently we constructed a whole-body mathematical model of cholesterol metabolism which was used to explore changes to both the rate of intestinal cholesterol absorption and the hepatic rate of clearance of LDL-C from the plasma. The model showed that of these two mechanisms, changes to the rate of LDL-C removal from the plasma with age had the most significant effect on cholesterol metabolism. The model was constructed using a series of coupled ordinary differential equations (ODEs). Additionally, the model was coded in the systems biology markup language (SBML) format and submitted to the Biomodels database to facilitate its updating and future exchange [17,18]. To build the model a wide variety of data was used including previously published data from a range of fields such as molecular biology, nutrition, physiology and biochemistry.

### Whole-body cholesterol metabolism

Cholesterol has a vital role to play in the human body. It is a key constituent of all cell membranes being involved in membrane fluidity; it is the precursor of steroid hormones which control a range of physiological functions, and bile salts, which are necessary for the intestinal absorption of cholesterol, fats and lipid soluble vitamins [19,20]. The mechanisms underpinning cholesterol metabolism interact to preserve the balance of cholesterol in the body. This balance is maintained by coordinated interactions between cholesterol absorption, excretion and synthesis.

### Intake, absorption and excretion

Cholesterol from both the diet and bile is taken up by the small intestine daily. Cholesterol absorption is an inefficient process and can vary significantly from person to person. The percentage of cholesterol absorbed in healthy subjects (both male and female) is in the range of 29-80% [21]. The heterogeneity in cholesterol absorption has been attributed to its complexity, which involves a large number of enzymes and transport proteins in a multi-step process, however the way these mechanisms interact to regulate absorption efficiency remains unknown [22]. It is clear that during digestion, bile acids are released from the liver and gall bladder into the intestine and as cholesterol is practically insoluble in aqueous environments, the bile acids serve to create lipid micelles [23]. The micelles are then transported to the brush border of jejunal enterocytes, where the cholesterol is transferred into the enterocytes. At this point the story becomes nebulous, as the mechanism(s) by which micellar cholesterol is absorbed through the brush border membranes independent of bile salt uptake remains a mystery. A long standing hypothesis suggests that cholesterol absorption occurs by passive diffusion down a concentration gradient [24,25]. However, recent evidence supports the hypothesis that protein facilitated mechanisms are involved in cholesterol uptake by the enterocyte [26,27]. When cholesterol is inside enterocytes, it has been suggested that it can be transported back to the intestinal lumen by a class of membrane proteins known as adenosine triphosphate binding cassette transporters. It has been proposed that these transporters may serve to efflux cholesterol from the enterocyte back into the intestinal lumen for excretion [28]. Although the precise mechanisms of cholesterol absorption are not yet fully understood, it is known that cholesterol not transported back to the intestinal lumen is esterified in the enterocyte by acyl-CoA-cholesterol acyltransferase (ACAT), assembled and packaged, together with triglycerides, into chylomicrons. Chylomicrons are just one of many lipid carriers, known as lipoproteins, found in the circulation of mammals. According to their density, lipoproteins are categorised as chylomicrons, very low density lipoproteins (VLDL), intermediate density lipoproteins (IDL), low density lipoproteins (LDL) and high density lipoproteins (HDL) [29]. Chylomicrons transport absorbed cholesterol via the lymph to the liver [30].

### The liver and whole-body cholesterol metabolism

The liver is the central organ involved in cholesterol metabolism. It is actively involved in the uptake of cholesterol from lipoproteins, is an important site for *de novo* synthesis and is capable of storing cholesterol as esters after esterification by ACAT. Furthermore, it is involved in the secretion of cholesterol-containing lipoproteins and is the sole organ capable of removing excess cholesterol from the body, either by secretion into bile or by conversion into bile acids [31]. Bile acids are synthesised from cholesterol in the liver and facilitate the solubilisation of cholesterol, a prerequisite for cholesterol absorption. Bile acids are released postprandially and will not solubilise dietary lipids unless above a critical concentration. The concentration gradient is generated by two factors. Firstly, bile acids are powerful acids that are impermeable to cell membranes. Secondly, the majority of bile acids are reabsorbed from the small intestine and return to the liver via the portal vein, where they are taken up by hepatocytes and re-secreted into bile [32]. The conservation of bile acids by active absorption from the small intestine results in a hepatic pool of bile acids that cycles several times with each meal. In humans, the bile acids circulate between six and 10 times per day, while around 400 mg of bile acids is lost daily through faecal excretion. A decreased return of bile acids to the liver is compensated for by increased *de novo* synthesis from cholesterol in order to maintain the bile salt pool [33]. Cholesterol not converted to bile acids or secreted into bile can be released from the liver into the circulation and transported to peripheral tissue. The liver secretes VLDLs and on entering the circulation VLDLs are hydrolysed by lipoprotein lipase (LPL) [34], leading to the formation of VLDL remnants and IDLs. IDLs are either taken up by the liver or further hydrolysed to LDLs, which are the main cholesterol carrier in the blood [35]. LDL is taken up by the liver or by peripheral cells, either independently or via the LDL-receptor (LDLR). Expression is high in the liver but LDLRs are also expressed in peripheral tissue [36,37]. Hepatic LDLR (HLDLR) expression is transcriptionally regulated in response to intracellular cholesterol levels [38].

### Reverse cholesterol transport

The flux of cholesterol from peripheral tissue to the liver is known as reverse cholesterol transport (RCT). This pathway plays a vital role in maintaining cholesterol balance, with its action resulting in the movement of cholesterol from peripheral tissue to the liver [39]. Consequently RCT presents the only route for excess cholesterol generated in peripheral tissue to be eliminated from the body. HDL plays a crucial part in RCT [40,41]. HDL synthesis takes place in the liver and intestine, commencing with the generation of nascent particles, small discoidal structures lacking cholesterol. HDL acquires free cholesterol from the cell membranes of several tissues, which is esterified by the action of lecithin:cholesterol acyltransferase (LCAT), resulting in cholesteryl ester-rich, mature HDL particles. The transport of high density lipoprotein cholesterol (HDL-C) to the liver may follow several routes. In the presence of the enzyme cholesteryl ester transport protein (CETP), a portion of cholesterol esters from the HDL particle can be redistributed to other lipoproteins (e.g. VLDL and LDL) in exchange for triglyceride [42]. This cholesterol is delivered back to the liver via the LDLR. An alternative route involves the mature HDL particle binding directly to scavenger receptors BI (SR-BI) on the liver [43].

### Cholesterol synthesis

Almost all the tissues in the body are capable of synthesizing cholesterol from acetyl-Co enzyme A (CoA) in a series of enzyme-mediated steps that are mainly restricted to the endoplasmic reticulum (ER). The major rate-controlling enzyme in this pathway is 3-hydroxy-3-methylglutaryl CoA (HMG-CoA) reductase. Cholesterol homeostasis is regulated in two ways, firstly, when the content of unesterified cholesterol in cells increases, the expression of the LDLR protein decreases. Furthermore, two key enzymes involved in cholesterol synthesis are repressed, thus, any subsequent increase in cellular cholesterol diminishes. When cholesterol levels fall, these pathways are reactivated [38,44,45].

### Dysregulation of cholesterol metabolism with age

The interaction between cholesterol absorption, synthesis, and excretion maintains whole- body cholesterol metabolism. A change to any of them can significantly influence the others. For example, numerous studies have shown that inhibition of cholesterol synthesis results in increased intestinal cholesterol absorption [46,47]. The close interaction between absorption and synthesis in maintaining cholesterol balance is consolidated by studies, which have shown that interference with cholesterol absorption is associated with increased cholesterol synthesis [47]. Dysregulation of cholesterol metabolism can lead to high levels of LDL-C, while population studies have consistently demonstrated that LDL-C rises with age in both males and females [7]. The reason for an increase in LDL-C in so many individuals across both genders remains unknown. Furthermore, the intrinsic mechanisms that contribute to the rise in LDL-C with age remain incomplete; however a picture is beginning to emerge of potential candidates. A significant finding was the discovery that there is a gradual reduction in the rate of clearance of LDL-C from the circulation with age [48-50]. Adding further to this is the evidence that the number of hepatic LDLRs (HLDLRs) diminish with age in certain species [10,50]. Additionally, it has been reported that there is an increase in intestinal cholesterol absorption efficiency with age in rats [8,51]. However, to date no single intrinsic mechanism or combination of intrinsic mechanisms has conclusively been attributed to the rise in LDL-C with age [9]. Consequently our whole-body mathematical model investigated the hypothesis that age-related changes to the rate of intestinal cholesterol absorption and the rate of removal of LDL-C from the plasma could be responsible for the rise in LDL-C with age in humans.

## Results

### Results overview

**Figure 1:**
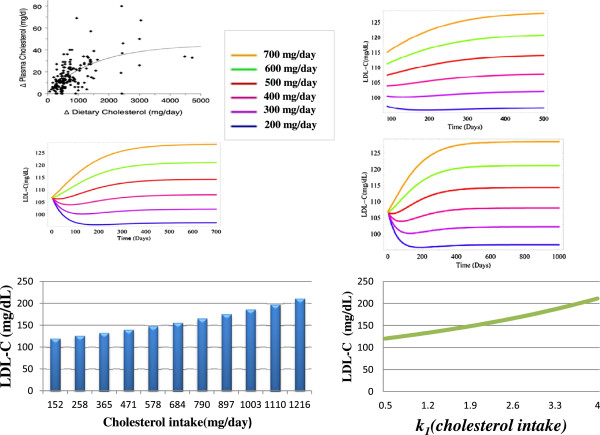
**(A-F)The response of the model to changes in the intake of dietary cholesterol compared to a published meta**-**analysis.A)** The relationship between mean change in dietary cholesterol (mg/day) and mean change in plasma cholesterol levels (mg/dL) in 167 cholesterol feeding studies published between 1960 and 1999 (McNamara 2000). **B**-**D)** The response of the model to changes in dietary cholesterol over a range of time periods. **E)** Steady-state levels of LDL-C for various different intakes of dietary cholesterol. **F)** The change in LDL-C levels in response to various different values of the parameter *k*_*1*_(cholesterol ingestion). Results of both E&F were generated from a sensitivity analysis of the model using the software tool Copasi.

Using the values listed in tables one and two, a sensitivity analysis was conducted, followed by a number of simulations that examined the key questions associated with this research. The model was able to replicate many of the integral features of whole-body cholesterol balance and displays qualitative behaviour similar to that observed in experimental studies [52]. For example, plasma LDL-C levels rose in response to increases in the intake of dietary cholesterol, however this was somewhat more sensitive than that predicted by a meta-analysis of the experimental literature, as for every 100mg/day increase in dietary cholesterol there was a ≈10mg/dL increase in LDL-C (Figure 1). Changes to cholesterol absorption were also investigated and the model showed that for every 10% increment in the rate of cholesterol absorption, this resulted in a 12.5 mg/dL increase in LDL-C. Finally, the model was used to investigate the hypothesis that age related changes to cholesterol absorption/a decrease in the clearance of LDL-C from the circulation result in the rise of LDL-C with age. The model showed that of these two mechanisms, changes to the rate of removal of LDL-C from the circulation had the most significant impact on LDL-C levels with age.

### Predictive capability of the model

**Figure 2:**
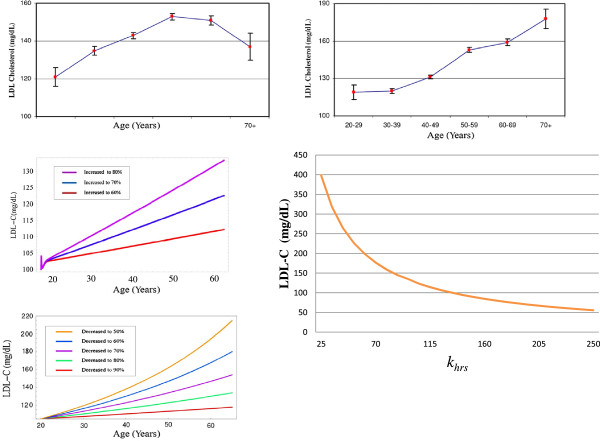
**(A-E)The relationship between LDL**-**C and age.** The relationship between LDL-C and age **A)** for males and **B)** for females. Graphs A and b were both generated using data from Abbott, Garrison et al. (1983). **C)** the response of LDL-C in the model to increased cholesterol absorption. **D)** The response of LDL-C in the model to decreases in the rate of clearance of LDL-C by hepatic LDLRs. **E)** Parameter scan for *k*_*hrs*_ (daily rate of synthesis of hepatic cholesterol receptors) and its impact on LDL-C levels.

The model demonstrated that increasing cholesterol absorption from 50% to 80% and running a simulation from age 20 years to age 65 years resulted in an increase of plasma LDL-C by as much as 34 mg/dL (Figure 2, graph A). However, reducing the number of hepatic LDL receptors had a profound impact on the system, as an increase of 116 mg/dL in LDL-C was witnessed by age 65 years in response to a reduction in the number of these receptors by 50% (Figure 2 graph B). These rises were compared to that found in population studies (Figure 2 graphs C and D) with changes to the rate of clearance of LDL-C clearly having the more significant impact on LDL-C levels.

### Sensitivity analysis-*cholesterol absorption*

**Figure 3:**
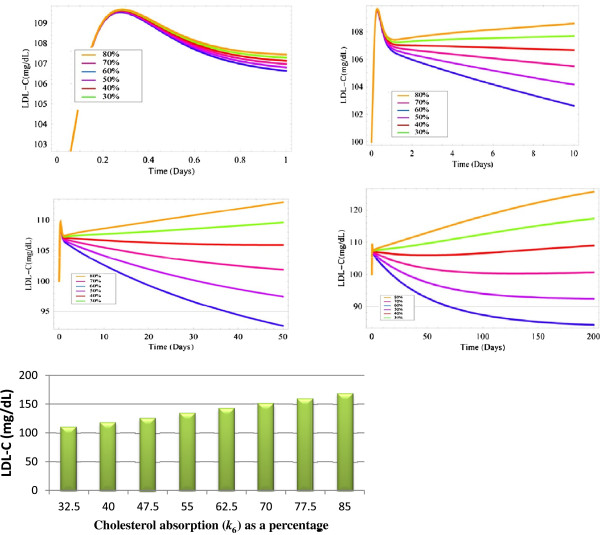
**(A-E) Changes to cholesterol absorption in the range 30-85%.A**-**D)** simulations of the model using MathSBML to show the response of LDL-C to changes in the rate of efficiency of cholesterol absorption in the range 30-80% over a number of different time periods. **E)** Steady-state levels of LDL-C for various different percentages of cholesterol absorption. This was generated with the software tool Copasi.

The initial concentrations of the various species are summarised in Table 1. Where quantitative data was available, these values reflected the numbers found in the literature. A sensitivity analysis of the model was also conducted as the parameter values differ considerably in terms of their magnitude (Table 2) and we wanted to establish how changes to these parameters would affect the concentrations of key species. Cholesterol absorption (*k*_*6*_) is an example of one of the key parameters that was altered. The initial value of this parameter was set so that 50% of cholesterol entering the small intestine would be absorbed. However, although cholesterol absorption is tightly regulated, it is a process that can vary significantly from one individual to the next [21]. This range was used to test the sensitivity of the model to changes in *k*_*6*_. The impact variations in cholesterol absorption has on LDL-C levels in the model is shown in (Figure 3).

**Table 1:** **List of species**, **their abbreviations and their initial values**

**Dietary Species**	**Symbol**	**Initial Value**
Dietary cholesterol	DC	304 mg
**Intestinal Species**		
Intestinal cholesterol	IC	3150 mg
Intestinal bile salts	IBS	467 mg
Intestinal cholesterol synthesis	ICS	0 (Source Species)
Intestinal nascent high density lipoprotein synthesis	INHDLS	0 (Source species)
**Excreted Species**		
Excreted cholesterol	EC	0
Excreted bile salts	EBS	0
**Hepatic Tissue Species**		
Hepatic cholesterol synthesis	HCS	0 (Source Species)
Hepatic nascent HDL synthesis	HNHDLS	0 (Source Species)
Hepatic bile salt pool	HBS	400 mg
Hepatic free cholesterol	HFC	60000 mg
Hepatic low density lipoprotein receptors	HLDLRs	100 (Theoretical value to represent the number of hepatic LDL receptors )
Hepatic low density lipoprotein receptor synthesis	HLDLRsS	600 (Source species)
Hepatic LDL receptors degradation	HLDLRD	0 (Sink species)
Hepatic cholesterol esters	HCE	10000 mg
Scavenger receptor class B type 1	SRB1	100 (Theoretical value to represent concentration of SRB1 receptors)
**Peripheral Tissue Species**		
Peripheral low density lipoprotein receptors	PLDLRs	100 (Theoretical value)
Peripheral low density lipoprotein receptors synthesis	PLDLRsS	575.16 (Source species)
Peripheral low density lipoprotein receptors degradation	PLDLRD	0 (Sink species)
Peripheral free Cholesterol	PFC	57516 mg
Peripheral cholesterol esters	PCE	9363 mg
Peripheral steroid synthesis	PSS	0 (Sink species)
Peripheral cholesterol synthesis	PCS	0(Source species)
**Plasma Species**		
Low density lipoprotein cholesterol	LDLC	100 mg/dL
High density lipoprotein cholesterol	HDLC	45 mg/dL
Nascent high density lipoprotein	NHDL	100 (Theoretical value to represent the initial number of Nascent HDL)
Very low density lipoprotein cholesterol	VLDLC	20 mg/dL
Intermediate density lipoprotein cholesterol	IDLC	20 mg/dL
Cholesteryl ester transfer protein	CETP	100 (Fixed boundary condition)
Lecithin:cholesterol acyltransferase	LCAT	100 (Fixed boundary condition)
Hormone sensitive Lipase	HSL	100 (Fixed boundary condition)
Lipoprotein Lipase	LPL	100 (Fixed boundary condition)
**Hepatic Tissue Species and Peripheral Tissue**		
**Species**		
Cholesterol ester hydrolase	CEH	100 (Fixed boundary condition)
acyl coenzyme A: cholesterol acyltransferase	ACAT	100 (Fixed boundary condition)

**Table 2:** Summary of the reactions and parameter values used in the model

**Reaction**	**Parameter**	**Value**	**Units**
Cholesterol intake	*k*_1_	1	mg/day
Bile salt release	*k*_2_	6	mg/day
Hepatic return of bile salts	*k*_3_	4.29	mg/day
Bile salt excretion	*k*_4_	8.56 × 10^-1^	mg/day
Bile salt synthesis	*k*_5_	2.66	mg/day
Cholesterol absorption	*k*_6_	5.29 × 10^-4^	mg/day
Cholesterol excretion	*k*_7_	5.29 × 10^-4^	mg/day
Intestinal Nascent HDL synthesis	*k*_8_	5 × 10^-4^	mg/day
Hepatic cholesterol storage	*k*_9_	1	mg/day
Release of stored Hepatic cholesterol	*k*_10_	5.998	mg/day
Hepatic Nascent HDL Synthesis	*k*_11_	5 × 10^-2^	mg/day
VLDL cholesterol formation	*k*_12_	1.6 × 10^-2^	mg/dL/day
Synthesis of hepatic LDL receptors	*k*_*hrs*_	100	number/day
Hepatic LDL receptors degradation	*k*_13_	1 × 10^-3^	number/day
VLDL cholesterol hepatic reuptake	*k*_14_	4.96 × 10^-3^	mg/dL/day
IDL cholesterol formation	*k*_15_	4.3 × 10^-1^	mg/dL/day
IDL cholesterol hepatic reuptake	*k*_16_	5.4 × 10^-2^	mg/dL/day
LDL cholesterol formation	*k*_17_	3.8 × 10^-1^	mg/dL/day
Hepatic LDL receptors uptake of LDL-cholesterol	*k*_18_	6.80 × 10^-2^	mg/dL/day
Hepatic receptor independent uptake of LDL-cholesterol	*k*_19_	5.0 × 10^-3^	mg/dL/day
Peripheral LDL receptors uptake of LDL-cholesterol	*k*_20_	6.75 × 10^-3^	mg/day/day
Peripheral independent uptake of LDL-cholesterol	*k*_21_	5.0 × 10^-6^	mg/dL/day
Synthesis of peripheral LDL receptors	*k*_*prs*_	100	number/day
Peripheral LDL receptors degradation	*k*_22_	1 × 10^-2^	number/day
Peripheral cholesterol storage	*k*_23_	1.75 × 10^-2^	mg/day
Release of stored peripheral cholesterol	*k*_24_	1.07 × 10^-1^	mg/day
Peripheral steroid production	*k*_25_	5 × 10^-4^	mg/day
HDL cholesterol formation	*k*_26_	1.5 × 10^-5^	mg/dL/day
CETP mediated transfer of cholesterol to VLDL from HDL	*k*_27_	10 × 10^-3^	mg/dL/day
CETP mediated transfer of cholesterol to LDL from HDL	*k*_28_	1 × 10^-3^	mg/dL/day
Reverse cholesterol transport	*k*_29_	5.0 × 10^-2^	mg/dL/day
Maximum rate of intestinal cholesterol synthesis	ICS_max_	1 × 10^2^	mg/day
Intestinal cholesterol synthesis threshold	ICS_t_	3.120 × 10^2^	mg/day
Sensitivity of intestinal cholesterol synthesis	IS	5	Fitting parameter
Maximum rate of biliary cholesterol release	BCR_max_	2 × 10^3^	mg/day
Biliary cholesterol release threshold	BCR_t_	5.55 × 10^4^	mg/day
Sensitivity of the feedback equation for biliary cholesterol	BS	5	Fitting parameter
Maximum rate of hepatic cholesterol synthesis	HCS_max_	5 × 10^2^	mg/day
Sensitivity of hepatic cholesterol synthesis	HS	5	Fitting parameter
Hepatic cholesterol synthesis threshold	HCS_t_	9.39 × 10^4^	mg/day
Maximum rate of peripheral cholesterol synthesis	PCS_max_	5 × 10^2^	mg/day
Peripheral cholesterol synthesis threshold	PPC_t_	8.0342 × 10^4^	mg/day
Sensitivity of peripheral cholesterol synthesis	PCSS	5	Fitting parameter

### Sensitivity analysis-*dietary cholesterol*

A literature review using PubMed revealed the existence of almost 200 publications containing information relating to the response of plasma cholesterol to dietary cholesterol feeding. These investigations were performed in both young and old individuals. Importantly among these publications, a meta-analysis by McNamara (2000) revealed that in response to increased levels of dietary cholesterol, plasma cholesterol plateaus at high levels of cholesterol intake [52]. The meta-analysis by McNamara also reported that the total plasma cholesterol response to dietary cholesterol is 0.023 mg/dL per mg/day cholesterol increase (Figure 1, graph A). Of this, 0.019 mg/dL is in the LDL-C fraction. Therefore, for each 100 mg/day increase in dietary cholesterol intake in the model, a 1.9 mg/dL increase in LDL-C was expected. Importantly, McNamara reported that this increase is independent of other types of lipid in the diet and the baseline plasma cholesterol level. Therefore, the model was exposed to a decrease of 200 mg/day and increases of up to 700 mg/day of dietary cholesterol (Figure 1, graphs A-C). A ≈10 mg/dL increase in LDL-C was observed for every 100mg increase in dietary cholesterol which was 8mg greater than the value predicted in the meta-analysis by McNamara. We also conducted a sensitivity analysis of the parameter *k*_*1*_ to highlight the different steady-state levels of LDL-C for various intakes of dietary cholesterol (Figure 1, graphs E&F and Additional file 1). No increase in HDL-C was observed in response to changes in dietary cholesterol.

### Hypothesis testing- *ageing and cholesterol absorption*/*LDL*-*C plasma removal*

Aging results in a rise in LDL-C in both sexes (Figure 2, graphs C and D) [7]. The reason(s) for an increase in LDL-C in so many individuals across both genders remains unknown and the issue is the subject of debate as nutritional status and physical activity levels may differ within the population sample. The intrinsic mechanism(s) that contribute to the rise in LDL-C with age remain incomplete; however a picture is beginning to emerge of potential candidates. As mentioned previously, rodent studies have shown that cholesterol absorption efficiency increases markedly with aging [8,21]. It was also mentioned that there is a gradual reduction in the rate of clearance of LDL-C from the circulation with age in rodents. Adding further to this is the evidence that the number of hepatic LDLRs diminish with age in certain species [29]. When cholesterol absorption efficiency was increased in 10% increments between 50% and 80% by age 65 years, 12, 24 and 34mg/dL increases respectively, in LDL-C were observed (Figure 2, graph A). Next, reducing the number of hepatic LDL receptors (HLDLRs) was investigated. The number of HLDLRs was reduced in the range 90–50 by age 65 years. This was done by gradually decreasing the rate of synthesis *k*_*hrs*_. This had a significant effect on the model as LDL-C raised by 116,76,49,26 and 11mg/dL respectively (Figure 2, graph B). A parameter scan of *k*_*hrs*_ was also conducted (Figure 2, graph E).

## Discussion

We have constructed a whole-body mathematical model of cholesterol metabolism using data from a wide variety of sources and integrated this data within a series of coupled ODEs. Previous computational models have focused on the intracellular regulation of cholesterol metabolism [16,53] or have focused on the metabolic fate of a particular lipoprotein or their receptor mediated endocytosis [54,55], while other mathematical models of lipid metabolism have centred on compartmental approaches, where by lipoproteins are represented by compartments [56]. Our model attempts to include all the major components of whole-body cholesterol balance and to our knowledge is the first model of its type built using such a framework. Our model provides an insight into the complex interplay of cholesterol metabolism with the aging process. The model has many areas that require further development, however, when the model progresses in the future it would be worthwhile to investigate how changes to different combinations of parameters affect the overall behaviour of the system, as the model indicates that the dysregulation of cholesterol metabolism with age may involve perturbations to several components of this system. In tandem with investigating changes to combinations of parameters, the model may also be used to investigate combination therapy that may help to mitigate the effects aging has on cholesterol metabolism. For example, it would be straightforward to include hypothetical interventions that investigate different dietary regimes and also include the effects of variations in physical activity. For example, based on available literature the simple assumption could be made that consuming 3 g/day of fibre reduces cholesterol absorption by 15% [57,58]. Therefore, the parameters could be adjusted accordingly to reflect this. Similarly the effect of consuming plant sterols, which are known to reduce cholesterol absorption [59,60] could easily be investigated, as consumption of 1.8 to 2.0 g/day of plant sterols has been shown to lower both total and LDL-C concentrations by 10% to 15%, respectively in a variety of different population groups [61-63]. Thus, this could be investigated by making alterations to the rate of cholesterol absorption in the model.

As mentioned previously the model is by no means the finished product and several assumptions were made during the building of the model, which were necessary as considerable uncertainty still surrounds cholesterol metabolism. For example, in the majority of cases the functional form that best describes a reaction remains unknown. We did however include a number of feed-back and feed-forward equations to describe the behaviour of cholesterol and bile salt synthesis as these are known to be subject to this type of action. At steady state, parameter values attempted to reflect a generic normolipididemic 20 year old male. Although parameter values were chosen for the model from available published literature, the ranges of parameter values are quite broad; however, given the nature and diversity of the literature used this was unavoidable. On certain occasions, parameter values simply did not exist, therefore reasonable values were chosen based on the published literature. An area of the model that could be developed further in the future is the kinetic parameters of the various enzymes involved in a number of the reactions. Presently, most of these enzymes have arbitrary values of 100 assigned to them to indicate that they have ‘normal’ enzymatic activity. In the future it would be worthwhile to use a resource such as BRENDA to amend these enzymes to include *k*_m_ and *k*_cat_ values [64]. Despite these limitations it is hoped that in the future the model will prove to be a useful tool for testing nutritional and lifestyle interventions that would be difficult, challenging or perhaps unethical to conduct using convention means.

Building the model has highlighted priorities for future experimental work, for example the need for experimentalists to work closely with computational modellers in order to generate appropriate quantitative data that is of benefit to systems biology models such as this one. Such data could be used to expand and enhance our model leading to a better understanding of cholesterol metabolism, particularly the interrelationship between cholesterol synthesis and absorption. This improved model could be used to gain additional insights into the factors associated with cholesterol metabolism that help to confer increased longevity and healthy aging. Appropriate dietary intervention strategies based on such models could be used to decrease the risk of CVD and prolong healthy aging. Models could be tailored for an individual’s dietary, biochemical, genetic and social circumstances and as such models can be coded in SBML they would be relatively easy to extend, enhance and improve as more quantitative data becomes available. Furthermore, it would be worthwhile combining our model with existing models, such as that of atherosclerosis progression [65]. The portable nature of SBML also makes this a very realistic possibility, especially if models that are being merged have been designed using SBML [66,67]. Moreover, the model could be used to investigate crosstalk between cholesterol metabolism and fatty acid metabolism.

## Conclusions

From the evidence presented in this paper it is clear that cholesterol metabolism is a complex multi-component system. Changes to this multi-component system as a result of the ageing process can lead to high levels of plasma cholesterol particularly LDL-C. There is an indisputable link between elevated levels of LDL-C and the risk of developing atherosclerosis, with population studies indicating that LDL-C rises with age in both sexes in a large number of individuals [5,68,69]. It is therefore unsurprising that recent genetic studies have associated lipid metabolism with longevity as it is apparent that maintaining cholesterol balance is vital to cardiovascular health [1,2,70,71].

We developed a whole-body computational model to investigate the hypothesis that an increase in the rate of cholesterol absorption and a reduction in the rate of removal of LDL-C from the plasma were integral to understanding the dysregulation of cholesterol metabolism with age. We were of the opinion that existing models of cholesterol metabolism were limited and could not offer a sufficiently deep understanding of how an entire biological system changes with age, thus in this work a conscious effort was made to represent the interactions throughout the body. Results from the sensitivity analysis of the model indicated that the model requires further fine-tuning in the future before it could be used as a comprehensive tool for predicting changes to LDL-C in response to dietary cholesterol/fats and intrinsic aging. None the less we used the model to investigate aging and it demonstrated that changes to the rate of removal of LDL-C to half its original value at age 65 years resulted in a 116 mg/dL rise in this cholesterol sub fraction, which is intimately connected with CVD. The model contrasts with other models of cholesterol metabolism in that it is implemented in a whole-body fashion and provides a template for building a quantitative systems level understanding of cholesterol metabolism and its interaction with aging. In the future the model could contribute to a better understanding of cholesterol metabolism so that the wider population could benefit in the same way as those individuals with exceptional longevity have from an altered lipid profile.

## Methods

### Deciding on model structure and the rationale for a whole-body approach

Historically, mathematical modelling has been used to investigate various aspects of both cholesterol and more generally lipid metabolism; however to our knowledge no model to date has been implemented to investigate cholesterol metabolism mechanistically within a whole- body framework, with the goal of understanding how intrinsic age-related biological changes affect this crucial biological system. In order to appreciate the rationale underpinning the whole-body approach that we adopted, it is worthwhile examining some of the models of cholesterol metabolism that have been developed previously. Additionally, it is worth exploring why these were unsuitable for this work. In general these models can be divided into three types; intracellular models of the cholesterol homeostasis genetic regulatory pathway; models of lipoprotein dynamics, and compartmental models. If we take the gene network approach, a recent version involved a boolean network model that assigned 33 different components of intracellular cholesterol homeostasis a boolean value. Simulations then led to the representation of intracellular cholesterol homeostasis as a boolean vector with each coordinate of the system denoting a biological species of the pathway [16]. It is easy to appreciate the utility of this approach as a means of better understanding the functioning of a gene regulatory network; however this strategy is not a mechanistic approach. It is primarily a state based representation of intracellular gene activity; therefore it is limited as it does not include the different biological compartments involved in cholesterol metabolism, such as absorption, and hepatic/peripheral cholesterol synthesis. Nor does it account for the synthesis of the various lipoproteins such as VLDL, IDL, LDL and their subsequent interactions with one another and with receptors both hepatically and peripherally. Finally, and significantly for this work it is difficult to imagine such models being able to test a biological hypothesis that centres on three factors, the aging process, cholesterol absorption and the removal of LDL-C from the plasma.

If mathematical models focused on lipoprotein kinetics are examined; one discovers a wide variety of models that have represented varying degrees of cholesterol metabolism and have centred to a large extent on the dynamics of the LDL receptor. Examples of such models go back as far as the early 1980s when Goldstein et al. presented a theoretical study of the interaction of LDL receptors with coated pits [72,73]. These were followed by other models of the same process [73] or more recent slight adaptations of this process [74]. These models have been beneficial from the point of view of elucidating the underlying dynamics which describe the interaction of LDL with its receptor and the associated underlying kinetics of this process. However, as with the intracellular models, these models focus on one particular, isolated aspect of cholesterol metabolism and do not portray the entire picture which is that cholesterol metabolism involves the coordinated action of several different biological systems operating in unison throughout the entire body.

The final approach that will be discussed is the compartmental methodology. Compartmental models, as the name suggests, contain a number of compartments, each containing a well mixed material. Each compartment has a number of connections leading into it and out of it. Biological material can flow from one compartment to another, and it can be added from the outside through a source, or it can be removed through a sink. Such systems exchange material with each other following certain rules [75]. These models have advantages such as being able to examine in some detail the interaction of lipoproteins and their derivatives. The major limitation of these models is that they are not based on underlying biological mechanisms; rather exchange rates are based on experimental inferences. Therefore, as with the previous approaches this method is also unsuitable for representing a mechanistic whole-body of cholesterol metabolism.

In order to overcome the limitations of the approaches that have been discussed, it was decided to represent cholesterol metabolism with the aid of biological compartments that represented their real-world biological counterparts. The compartments contained quantitative details of the different forms of cholesterol. These forms are present in very high numbers (e.g. intestinal, hepatic and peripheral cholesterol is present in grams). This means that a stochastic approach would not offer significant benefits, as the intrinsic stochasticity in the system is negligible, and so a deterministic model was considered more appropriate to represent these species and their interactions. In order to implement this deterministic solution, we created a series of ordinary differential equations that were coupled and numerically integrated. This type of approach has previously been shown to be eminently suitable both for representing mechanistic biological reactions and for dealing with the time dimension associated with modelling the aging process [13]. In order to build the ODEs, we first needed to concisely represent the compartments and the fundamental components of whole-body cholesterol metabolism diagrammatically.

### Construction of a network diagram

**Figure 4:**
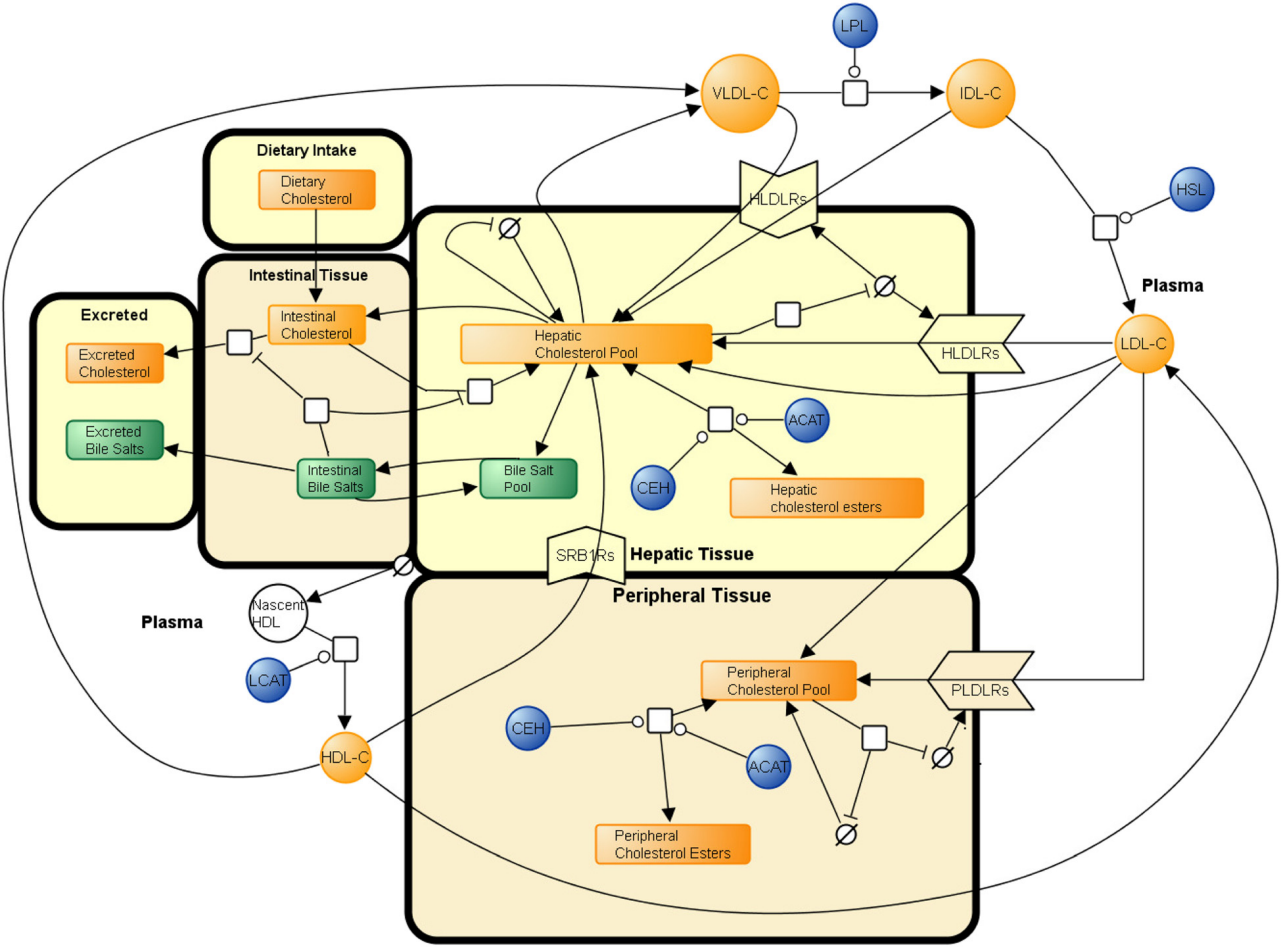
**Network diagram of the cholesterol metabolism model.** The model is laid out in 6 compartments, **1)** intake, **2)** intestinal tissue, **3)** excretion, **4)** plasma, **5)** hepatic and **6)** peripheral tissue. The arrows represent the flow of cholesterol around the system into its different forms. Enzymes are represented by blue spheres and their catalytic influence on the reaction is indicated by a round arrow head coming into contact with a reaction arrow. Synthesis is represented by the Greek symbol theta, while inhibition is represented by T -shaped arrows. In summary cholesterol from the diet and bile is formed into micelles in the small intestine. Absorbed cholesterol is then transported to the liver where it is exported into the plasma via VLDL. VLDL is in turn catabolised to LDL. Excess cholesterol from peripheral tissue is transferred to the liver via HDL.

Whole-body cholesterol metabolism is a difficult process to reason about directly; therefore the first step in constructing a model of this nature was to assemble a network diagram (Figure 4) using Systems Biology Graphical Notation (SBGN). SBGN is a standardized means of graphical representation of biological systems that facilitated the drafting of a network diagram of cholesterol metabolism in a clear and unambiguous fashion [76]. This network diagram explicitly outlines each of the compartments and the biological species involved in whole-body cholesterol metabolism which we have discussed. Each of the components of Figure 4 was then abbreviated and used to construct a series of ODEs (Table 1 contains a summary of these abbreviations).

### Model assembly

#### Rationale for reaction forms

The assumption was made that a large number of the reactions are of a first-order nature, where the rate of the reaction is directly proportional to the concentration of one of the reactants. This assumption was made to facilitate the evaluation of the model based on steady states and to allow for the determination of parameters when the model was compared to experimental data, moreover the reaction form that best describes a large number of these remains unknown. ODEs were constructed in an incremental fashion to meet certain steady states, eventually giving rise to a unified series of coupled ODEs and ultimately a whole-body mathematical model representing a generic twenty year old male. The model was built using MathSBML [77]. MathSBML is a software package that has been designed to work with Mathematica (version 5.2) [78], a pre-existing commercial software package developed to perform numeric, algebraic, graphical and many other tasks. In addition the software tool Copasi was used to perform some of the parameter scans and steady state examinations of the model [79]. The next section outlines the assembly of the ODEs and the biological functions that lie at their core. Additionally, the reasoning for the inclusion of each of the species in Figure 4 will be detailed along with the quantitative data that was used to make inferences about each of the steady states that these species gave rise to.

#### Cholesterol intake

As whole-body cholesterol metabolism was being dealt with, it was important first to include the dietary intake of cholesterol. According to Henderson et al. the average amount of cholesterol ingested daily in the UK by a male is 304 mg/day [80]. Cholesterol intake was assigned the rate *k*_1_ and set at an initial value of 1. While dietary cholesterol [DC] had its initial condition set to a fixed value of 304.

#### Cholesterol synthesis

**Figure 5:**
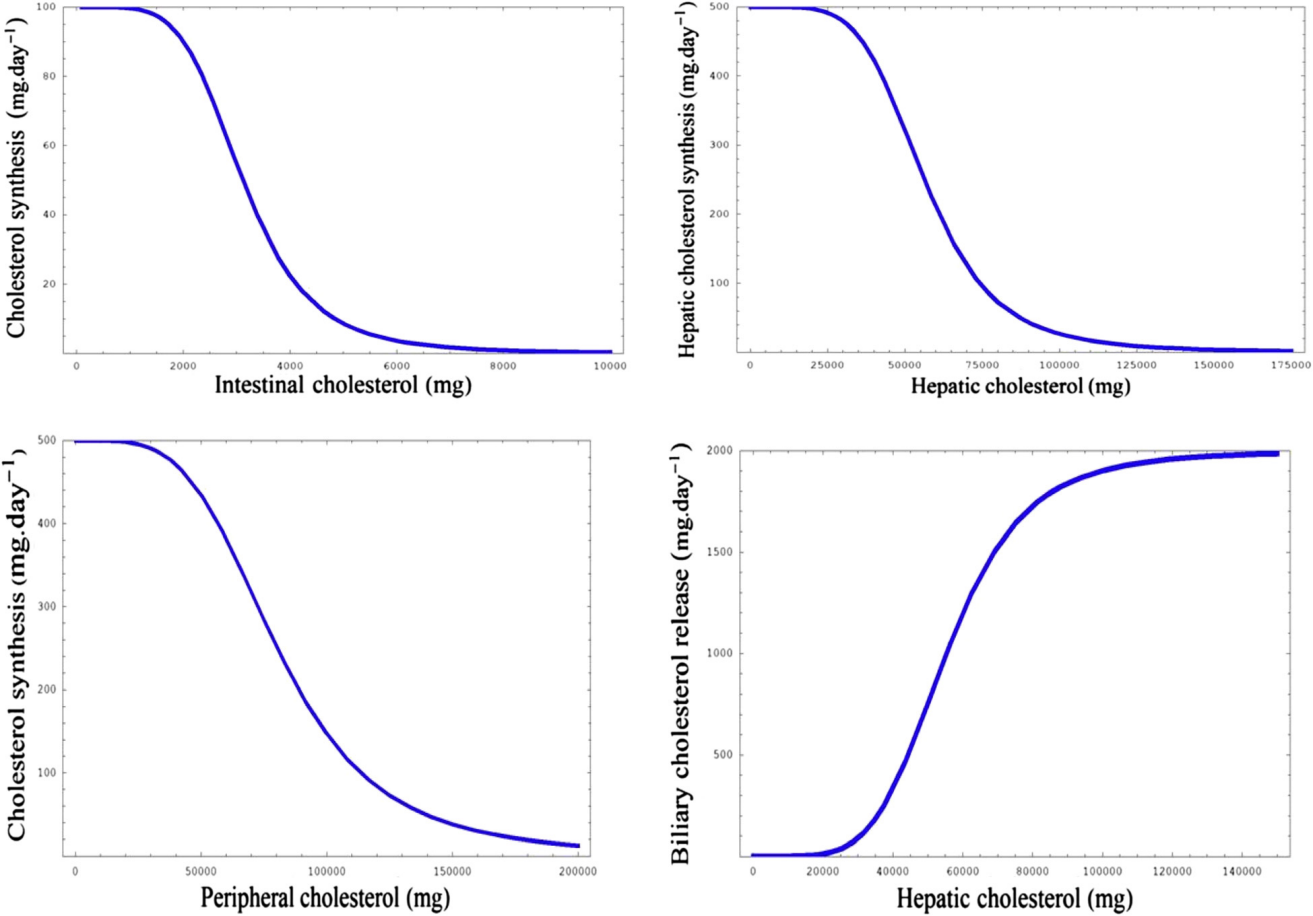
**(A-D)Functions and simulations associated with model building.A)** Graph of concentration of intestinal cholesterol versus cholesterol synthesis. **C** and **D)** Graphs of the functions for hepatic and peripheral cholesterol synthesis respectively. **D)** Graph of concentration of hepatic cholesterol versus biliary cholesterol release state values being reached for IDL-C and VLDL-C, respectively.

Dietary cholesterol joins synthesised intestinal cholesterol. Additionally, cholesterol is also synthesised throughout the body [81]. To determine the daily rates of synthesis in each compartment, whole-body cholesterol synthesis was first calculated. According to Dietschy et al. humans synthesise ≈10 mg/kg of cholesterol daily, therefore a 70 kg man synthesises ≈700 mg/day [81]. The amount of cholesterol synthesis that can be attributed to the liver, intestine and remaining peripheral tissue is uncertain [81]. Therefore, whole-body synthesis of cholesterol for a 70 kg man was based on the assumption that ≈70% of cholesterol synthesis takes place in the peripheral tissue and that 10% of this occurs in the intestine [82] (Table 3). This data was then used to make inferences about inter-compartmental cholesterol synthesis and functional relationships were derived to describe cholesterol synthesis in each compartment. Firstly, intestinal cholesterol synthesis was represented with a negative feedback function (equation 1) were ICS_max_ represents the maximum amount of cholesterol that can be synthesised by the intestine in a day, IC represents intestinal cholesterol. IC_t_ represents the intestinal cholesterol synthesis threshold and IS is a fitting parameter that represents the sensitivity of the negative feedback. It was assumed that the maximum rate of intestinal cholesterol synthesis would be 100 mg/day (Figure 5, graph A).

(1) $$\text{Intestinal cholesterol Synthesis}=\frac{ICS_{\max}}{1+{\left(IC/IC_{t}\right)}^{\mathrm{IS}}}\;$$

**Table 3:** **Breakdown of whole**-**body cholesterol synthesis**

**Compartment**	**Cholesterol synthesised**
Whole-Body (70 kg man)	700 mg/day
Peripheral Tissue	441 mg/day (70% of whole-body)
Intestine	49 mg/day(10% of peripheral)
Liver	210 mg/day

We also derived similar functions for both hepatic and peripheral synthesis (equations 2 &3, respectively). HCS_max_ and PCS_max_ represent the maximum amount of cholesterol that can be synthesised daily in the hepatic and peripheral compartments respectively. HCS_t_ and PPC_t_ represent hepatic and peripheral daily rates of synthesis thresholds respectively and HS and PCSS are fitting parameters that represent the sensitivity of the feedback in the two respective compartments. Based on experimental data it was assumed that the maximum daily rate of cholesterol synthesis in the liver would be 500 mg/day, while the maximum rate of peripheral cholesterol synthesis was also estimated to be 500 mg/day (Figure 5, graphs B and C).

(2) $$\text{Hepatic cholesterol synthesis=}\;\frac{HCS_{\max}}{1+{\left(HFC/HCS_{t}\right)}^{\mathrm{HS}}}$$

(3) $$\text{Peripheral cholesterol synthesis=}\frac{PCS_{\max}}{1+{\left(PFC/PPC_{t}\right)}^{\mathrm{PCSS}}}$$

#### Hepatic biliary cholesterol release

According to Grundy and Metzger dietary cholesterol also mixes intestinally with ≈ 1200 mg/day of biliary cholesterol [83]. Therefore, a functional relationship was derived to describe the release of biliary cholesterol. This was done using a feed-forward equation (equation 2) as by nature this biological interaction is feed-forward. BCR_max_ represents the maximum daily release of biliary cholesterol. BCR_t_ represents the threshold of release of biliary cholesterol and HFC represents the hepatic pool of free cholesterol. Based on experimental data it was assumed that the maximum rate of release of biliary cholesterol would be 2000 mg/day (Figure 5, graph D) [83].

(4) $$\text{Release of biliary cholesterol=}\frac{BCR_{\max}}{1+{\left(BCR_{t}/HFC\right)}^{\mathrm{BS}}}$$

#### Estimating the steady-state levels of cholesterol in each compartment

It was necessary to estimate the steady-state level of cholesterol in each compartment. According to Soars et al. in mammals this usually falls within the range of 100-200 mg/100 g of intestine. Also according to Soras et al. humans contain 30 g of intestine per kg of body weight [84]. The concentration of cholesterol in the intestine was determined based on several assumptions; firstly it was assumed that there is 150 mg of cholesterol/100 g of intestine and secondly the assumption was made that the hypothetical individual weighed ≈70 kg. Details of this calculation are provided in Table 4.

**Table 4:** **Calculating the steady**-**state level of intestinal cholesterol**

**The steady**-**state level of intestinal cholesterol**	
Weight of intestine	70*30=2100 g
Number of 100 g segments in intestine	2100/100=21
Total amount of cholesterol equals the number of 100 g segments times 150 mg	21*150 mg
Total	3150 mg

#### Bile salts- hepatic synthesis and enterohepatic circulation

Bile salts are synthesised from the hepatic pool of free cholesterol [HFC] at a rate of ≈400 mg/day, while the hepatic bile salt pool [HBS] contains bile salt in the region of ≈4000 mg. This pool is released on average four to six times per day into the small intestine. Thus, a total of ≈24000 mg of bile salts enter the small intestine daily. The synthesis of bile salts is subject to negative feedback as the bile salt pool increases. Based on this information a reciprocal type function was derived to represent the production of bile salts (equation 5). Where *k*_*5*_ is the rate constant for the hepatic production of bile salts.

(5) $$\text{Hepatic Production of Bile Salts=}\frac{k_{5}\left[\text{HFC}\right]}{\left[\text{HBS}\right]}$$

Almost all bile salts are reabsorbed (≈23600 mg/day) and return to the liver, while the remainder (≈400 mg/day) are excreted [85]. Bile salt release was assigned the rate constant *k*_*2*_, while the hepatic return of bile salts was assigned the rate constant *k*_*3*_. This rate was dependent on the concentration of intestinal bile salts [IBS] and the concentration of [IC]. The excretion of bile salts [EBS] was assigned the rate constant *k*_*4*_. Putting this information together, ODEs 1 and 2 were assembled to describe how hepatic and intestinal bile salts change with time.

(6) $$\begin{array}{l} \frac{d\left[HBS\right]}{dt}=\left(\frac{k_{5}\left[HFC\right]}{\left[HBS\right]}\right)+k_{3}\left[IBS\right]\left[IC\right]\\ \;-k_{2}\left[HBS\right]\;\left(\text{ODE}\;1\right) \end{array}$$

(7) $$\frac{d\left[IBS\right]}{dt}=k_{2}\left[BSP\right]-k_{3}\left[IBS\right]\left[IC\right]-k_{4}\left[IBS\right]\;\left(\text{ODE}\;2\right)$$

#### Cholesterol absorption and excretion

The rate of cholesterol absorption and excretion depend on the concentration of intestinal bile salts. Over the years there has been controversy as to whether there is a difference between the absorption of dietary cholesterol and endogenously derived cholesterol. It is now accepted that they form an indistinguishable intestinal pool [54]. An assumption was made based on experimental data that 50% of cholesterol in the small intestine whether it originates from *de novo* synthesis, diet or bile will be absorbed in a normal individual each day while 50% would be excreted [21,86]. Table 5 presents a summary of the estimated flux of cholesterol into and out of the small intestine on a day to day basis. The daily rates of cholesterol absorption and excretion were represented by the rate constants k_6_ and k_7_ respectively, and thus we were able to derive ODEs 3 and 4 that represent the change with time of *IC* and excreted cholesterol *EC*, respectively.

(8) $$\begin{array}{l} \frac{d\left[IC\right]}{dt}=k_{1}\left[DC\right]+\left(\frac{BCR_{\max}}{1+{\left(BCR_{t}/HFC\right)}^{\mathrm{BS}}}\right)\\ \;-k_{6}\left[IBS\right]\left[IC\right]\;-k_{7}\left[IBS\right]\left[IC\right]\\ \;+\left(\frac{ICS_{\max}}{1+{\left(IC/IC_{t}\right)}^{\mathrm{IS}}}\right)\;\left(\text{ODE}\;3\right) \end{array}$$

(9) $$\frac{d\left[EC\right]}{dt}=+k_{7}\left[IBS\right]\left[IC\right]\;\left(\text{ODE}\;4\right)$$

**Table 5:** Flux of cholesterol into and out of the small intestine on a daily basis

**Contribution**	**Amount in mg**/**day**
Diet	304
Biliary cholesterol	1200
Intestinal synthesis	49
Total	1553
*Absorbed*	≈ *776*.*5*
*Excreted*	≈ *776*.*5*

#### Simulations of the mini model

**Figure 6:**
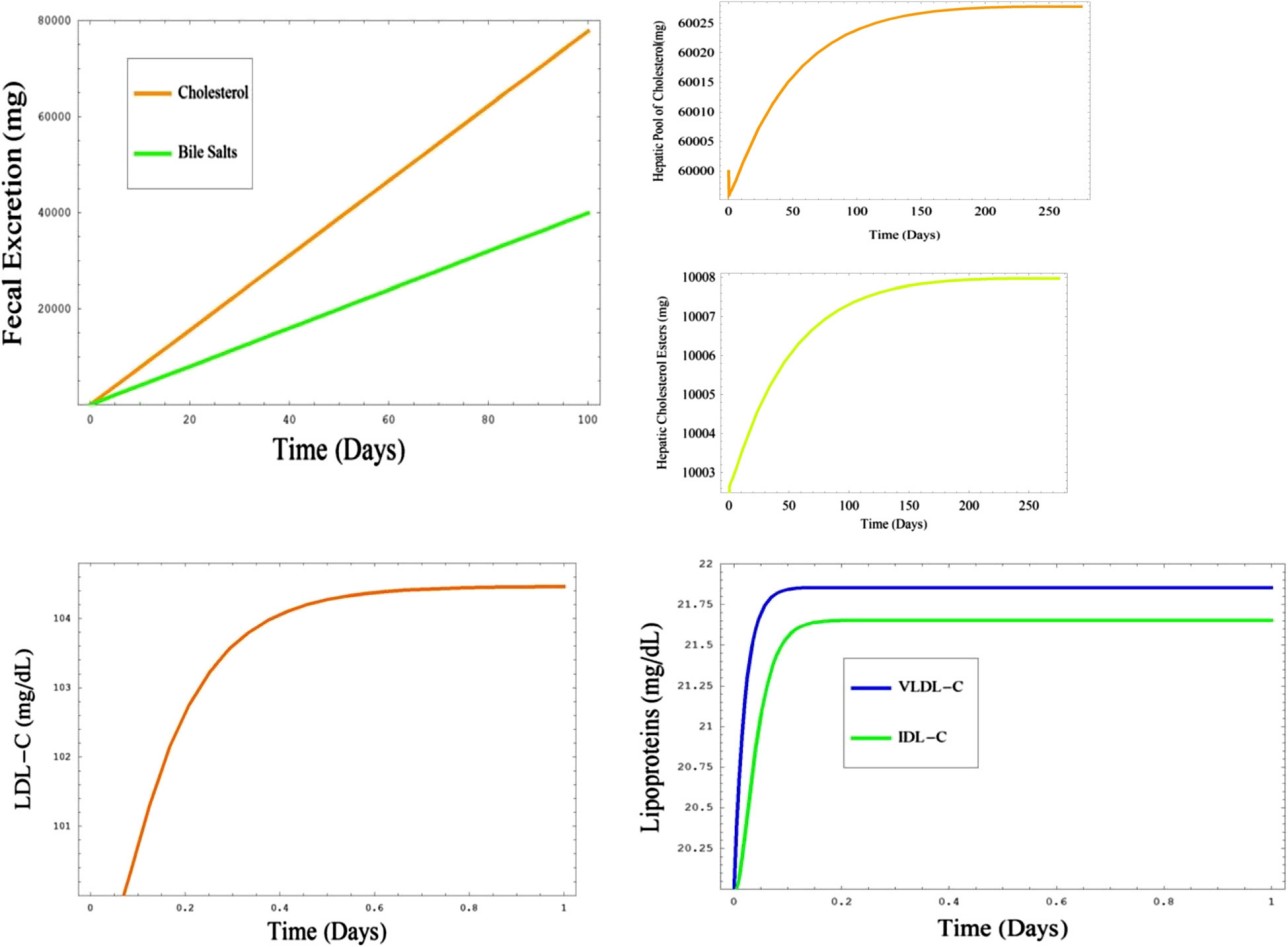
**(A-E)Outputs and steady states associated with model building.A)** Graph of the output from a simulation of the mini model that was constructed initially. It shows the faecal excretion of both cholesterol and bile salts over a 100 day period. **B** and **C)** Graphs showing the steady state values being reached for both the hepatic cholesterol pool and hepatic cholesterol esters respectively. **D** and **E)** Graphs showing steady state levels of LDL-C, IDL-C and VLDL-C.

At this stage it was critical to create a mini-model, as determination of parameter values becomes increasingly complicated as models gain in size and complexity. The parameters for the mini model were determined using the baseline data outlined and the ODEs were numerically solved using MathSBML. Figure 6, graph A, shows the amount of cholesterol and bile salts excreted over a 100 day period. This output from the mini-system confirmed that the intestinal and intake compartments, and part of the hepatic compartment were behaving in a biologically realistic manner, thus further compartments and species were added to the system. It is important to note that the hepatic pool of cholesterol was fixed at a value of 100 during these simulations to facilitate parameterisation.

#### Expansion of the mini model- further additions to the hepatic compartment

It was now necessary to estimate the steady state level of hepatic cholesterol. The amount of cholesterol in the liver was determined using data from Sahlin et al., the molecular weight of cholesterol and the weight of an average human liver [87]. Calculations are outlined in Table 6. According to Cook humans weighing ≈70 kg contain ≈ 140 g of cholesterol [88]. Based on the calculations in Table 6 the assumption was made that half the cholesterol in man resides in the liver, while the remainder resides in peripheral tissue. The assumption was also made that 10 g of cholesterol is stored as esters in each compartment. The hepatic synthesis of cholesterol was previously estimated to be 210 mg/day. When the hepatic pool of cholesterol increases, there is a rise in the conversion of cholesterol to esterified cholesterol. This reaction is catalysed by ACAT and the forward reaction was assigned the rate constant *k*_*9*_. The reverse reaction is catalysed by cholesterol ester hydrolases (CEH) and was assigned the rate constant *k*_*10*_. Both ACAT and CEH were given fixed vales of 100 to represent ‘normal’ enzymatic activity. Thus, based on this information we were able to derive ODEs 5 and 6 to describe the change in both the [HFC] and hepatic cholesterol esters [HCE] with time.

(10) $$\begin{array}{l} \frac{d\left[HFC\right]}{dt}=k_{19}+\left(k_{10}\left[HCE\right]\left[CEH\right]\right)\\ \;-\left(\frac{BCR_{\max}}{1+{\left(BCR_{t}/HFC\right)}^{\mathrm{BS}}}\right) \end{array}$$

(11) $$-k_{12}\left[HFC\right]-\left(\frac{k_{5}\left[HFC\right]}{\left[BSP\right]}\right)-k_{9}\left[HACAT\right]\left[HFC\right]$$

(12) $$\left(\frac{HCS_{\max}}{1+{\left(HFC/HCS_{t}\right)}^{\mathrm{HS}}}\right)\;\left(\text{ODE}\;5\right)$$

(13) $$\begin{array}{l} \frac{d\left[HCE\right]}{dt}=k_{9}\left[ACAT\right]\left[HFC\right]\\ \;-k_{10}\left[HCE\right]\left[CEH\right]\;\left(\text{ODE}\;6\right) \end{array}$$

**Table 6:** **Outlining how the concentration of cholesterol in the liver was determined** (**Non obese subjects** (**n**=**19)**)

**Liver sample**	**Liver homogenate**	**Liver microsomes**	**Total**
Mean free cholesterol	42.4 nmol/mg	24.7 nmol/mg	67.1 nmol/mg
Mean esterified cholesterol	9.9 nmol/mg	8.6 nmol/mg	18.5 nmol/mg
		Total Hepatic Cholesterol	115.6 nmol/mg
Molecular weight of cholesterol	386.66	Used to convert nmol to milligrams	
∴	Total cholesterol		44.6979 μg/mg or 0.0446979 mg/mg
	Total esters		7.1539 μg/mg or 0.0071532 mg/mg
∴	Total free cholesterol		37.5447 μg/mg or 0.0375447 mg/mg
Size of Human Liver = 1.5 kg [89]	1.5kg=1500000 mg		
	0.0446979 *1500000=67046.85 mg total cholesterol		
	0.0071532 * 1500000=10792.80 mg of cholesterol esters		
	0.0375447*1500000=56317.5 mg of free cholesterol		

These ODEs were added to the system and the model was re-parameterised by sequential adjustment of the parameters in order to bring both hepatic cholesterol esters and the hepatic pool of free cholesterol into a steady-state (Figure 6, graphs B and C).

#### Hepatic release of very low density lipoprotein cholesterol

According to Glomset et al. the amount of cholesterol secreted hepatically in VLDL is considered to be in the region of 800 mg/day and could be as high as 3000 mg/day [90]. Therefore, it was assumed that in the steady state ≈1000 mg/day of cholesterol was incorporated into VLDL. The rate of incorporation of cholesterol into VLDL was assumed to be proportional to the concentration of hepatic cholesterol and this reaction was assigned the rate constant *k*_12_.

#### The turnover of hepatic LDL receptors

According to Dietschy, the rate of lipoprotein removal from the circulation by the liver is dependent on the concentration of the lipoprotein and hepatic LDL receptors (HLDLRs) [82]. Brown and Goldstein established that the synthesis of HLDLRs is subject to inhibition by the intracellular concentration of free cholesterol [44,91]. Thus represented the synthesis of HLDLRs with equation 6, where *k*_hrs_ is the rate constant for the synthesis of HLDLRs.

(14) $$\begin{array}{l} \text{Synthesis of hepatic LDL receptors}\\ \;=\frac{k_{\mathrm{hrs}}\left[HLDLRs\right]}{\left[HFC\right]} \end{array}$$

It is challenging to determine the actual number of HLDLRs present on a human organ such as the liver. Although, the number has been estimated at a cellular level, it was not feasible to try and determine it for a whole-body model of cholesterol metabolism. Thus, an arbitrary value of 100 was assigned to represent a fully active complement of HLDLRs and the rate constant *k*_*13*_ was assigned to represent the rate of HLDLRs degradation. Thus, ODE 7 was derived to describe the turnover of HLDLRs. It is important to note that this type of approach has been used previously to model this process, whereby the regulation of receptor synthesis is handled via reciprocal feedback due to cholesterol concentration [92].

(15) $$\begin{array}{l} \frac{d\left[\text{HLDLRs}\right]}{dt}=\frac{k_{\text{hrs}}\left[\text{HLDLRs}\right]}{\left[HFC\right]}\\ \;-k_{13}\left[\text{HLDLRs}\right]\;\left(\text{ODE}\;7\right) \end{array}$$

It is known that a certain amount of VLDL-C is removed via *HLDLRs*[82]. It was difficult to obtain quantitative data about this process; however the literature indicated that this is not the major route by which cholesterol re-enters the liver. It was therefore assumed that only a negligible amount of VLDL-C is taken up via this avenue (≈100 mg/day) and was assigned the rate constant *k*_14_.

#### Hepatic LDL receptor mediated removal of VLDL-C from the plasma

The enzyme LPL acts on VLDLs to reduce them to IDLs [93]. It was assumed that this process is dependent on the concentration of LPL and of VLDL-C and was assigned the rate constant *k*_15_. Thus, ODE 8 was derived to represent the change with time of VLDL-C.

(16) $$\begin{array}{l} \frac{d\left[VLDLC\right]}{dt}=k_{12}\left[HFC\right]-k_{14}\left[\text{HLDLRs}\right]\left[\text{VLDLC}\right]\\ \;-k_{15}\left[\text{LPL][VLDLC] (ODE}\;8\right) \end{array}$$

Similar to VLDL-C, a certain amount of IDL-C re-enters the liver via HLDLRs and the assumption was made that this is ≈ 10% of the overall particle composition. IDLs are further catabolised to LDLs by the enzyme hormone sensitive lipase (HSL). The majority of LDL-C (≈ 75%) is taken up by the liver, while the remainder is removed by peripheral tissue. Based on the literature it was assumed that receptor independent uptake of LDL-C is of minor importance and accounts for only 50 mg/day of LDL-C removed from the tissue [94-96]. This value was split equally between the liver and peripheral tissue. Based on this information ODEs 9 and 10 were derived to describe the change in IDL-C and LDL-C with time. *k*_*16*_ is the rate constant for removal of IDLC via the hepatic receptor and *k*_*17*_ is the rate constant for the formation of LDL-C, while *k*_*18*_ is the rate constant for HLDLR uptake of LDL-C and *k*_*19*_ is the rate constant for the hepatic receptor independent uptake of LDL-C. Peripheral LDL receptor (PLDLRs) and non-peripheral receptor uptake are represented by the rate constants *k*_*20*_ and *k*_*21*,_ respectively.

(17) $$\begin{array}{l} \frac{d\left[IDLC\right]}{dt}=k_{15}\left[LPL\right]\left[VLDLC\right]-k_{16}\left[HLDLR\right]\left[IDLC\right]\\ \;-k_{17}\left[HSL\right]\left[IDLC\right]\;\text{(ODE}\;9) \end{array}$$

(18) $$\begin{array}{l} \frac{d\left[LDLC\right]}{dt}=k_{17}\left[HSL\right]\left[IDLC\right]-k_{18}\left[LDLC\right]\left[HLDLRs\right]\\ \;-k_{19}\left[LDLC\right]-k_{20}\left[LDLC\right]\left[PLDLRs\right]\\ \;-k_{21}\left(\text{ODE}\;10\right) \end{array}$$

#### Parameterisation to establish the steady states of LDL-C, IDL-C VLDL-C

As the system had now increased in size significantly, further simulations were completed to establish the steady-state levels of the various species. Again the model was re-parameterized using the data outlined and the system numerically solved with MathSBML. After adjustment of the parameters LDL-C, IDL-C and VLDL-C entered steady states based on known literature values (Figures 6, graphs D and E). It is important to note that the values of both the peripheral pool of free cholesterol (PFC) and PLDLRs were held at fixed arbitrary values of 100 to facilitate this process.

#### Further additions to the peripheral compartment

Once the first three compartments demonstrated reliable biological outputs, the final phase of model building involved the addition of the peripheral compartment. Table 7 outlines how the amount of cholesterol in the peripheral tissue was calculated based on previous calculations for the amount of cholesterol in the liver and intestine. Previously, the rate of peripheral cholesterol synthesis was estimated to be 441 mg/day. This was completed in exactly the same manner as for the hepatic pool, where the rate constants *k*_*prs*_ represents the synthesis of PLDLRs, *k*_*22*_ represents the degradation of PLDLRs, *k*_*23*_ represents the formation of peripheral cholesterol esters (PCE) and *k*_*24*_ is the release of stored peripheral cholesterol. Additionally, according to Myant, a certain amount of cholesterol can be converted to steroid hormones. For example, a normal man may excrete 25-50 mg/day of total adrenocortical and gonadal hormones [62]. As this loss has to be replaced, this was included in the model by the addition of the rate constant *k*_*25*_ which represents steroid hormone synthesis (PSS), which was in essence a sink species. ODEs 11 and 12 were then derived. Again at this point the model was re-parameterised and steady-states produced.

(19) $$\begin{array}{l} \frac{d\left[PCE\right]}{dt}=k_{23}\left[ACAT\right]\left[PFC\right]\\ \;-k_{24}\left[\text{PCE}\right]\left[\text{CEH}\right]\;\left(\text{ODE}\;11\right) \end{array}$$

(20) $$\begin{array}{l} \frac{d\left[PFC\right]}{dt}=k_{21}-k_{25}+k_{24}\left[PCE\right]\left[CEH\right]\\ \;-k_{23}\left[ACAT\right]\left[PFC\right]\frac{PCS_{\max}}{1+{\left(PFC/PFC_{t}\right)}^{\mathrm{PCSS}}}\\ \;+k_{20}\left[LDLC\right][PLDLRs]\;\text{(ODE}\;12) \end{array}$$

**Table 7:** Calculation of cholesterol in the peripheral tissue

**Tissue**	**Concentration**
Liver	70000 mg
Intestine	3120 mg
Remaining cholesterol in peripheral tissue	70000-3120= 66880 mg
Liver	10000 mg of stored cholesterol, which represents 14% of overall hepatic cholesterol
Peripheral tissue	Assuming 14% is stored here: 14% of 66880=3963.2 mg
Peripheral free cholesterol	66880-9363.2=57516.8 mg

#### Reverse cholesterol transport and the final steps to a whole-body mathematical model

Based on current literature it was assumed that the production of HDL-C is dependent on three factors; the concentration of peripheral free cholesterol, the population of nascent HDL particles and the activity of the enzyme LCAT which has a role to play in the conversion of free peripheral cholesterol into cholesteryl esters which are then sequestered into the core of a lipoprotein particle, eventually making the newly synthesized HDL particle. The enzyme is bound to HDLs and LDLs in the blood plasma.

Firstly, the population of nascent HDLs had to be represented. Nascent HDLs are synthesised in the intestine. As extensive literature searches revealed little quantitative information related to this process, an arbitrary value of 100 mg/day was assigned for the synthesis of nascent HDL. This value was then split evenly between its two points of origin; the intestine and liver. ODE 13 was then derived where *k*_8_ represents the intestinal rate of nascent HDL (*NHDL*) synthesis and *k*_11_ represents the hepatic synthesis of *NHDL*.

(21) $$\frac{d\left[NHDL\right]}{dt}=k_{8}+k_{11}\;\left(\text{ODE}\;13\right)$$

Reverse cholesterol transport is important as it represents the only route for excess cholesterol generated in the peripheral tissue to be removed from the body, either by secretion into bile or by conversion into bile acids [55]. In the steady state, RCT should equal the rate of synthesis of cholesterol in the peripheral tissue [41]. Previously the synthesis of cholesterol in the peripheral tissue was calculated to equal 441 mg/day. Therefore, 441 mg of cholesterol that originated in peripheral tissue should enter the liver via HDL and this return of cholesterol via HDL-C to the liver may follow one of several routes. In the presence of CETP, a portion of the HDL cholesterol is transferred to either VLDL or LDL and ultimately returned to the liver via HLDLRs*;* therefore it was assumed that 10% of the cholesterol that is scavenged by HDL-C makes its way to the liver via this path. Alternatively, HDL-C is delivered to the liver via scavenger receptor class B1 [SRB1]. Thus based on this information the change in HDL-C with time was represented by ODE 14, where *k*_*26*_ is the rate constant for the enzymatic dependent scavenging of cholesterol from the peripheral compartment, *k*_*27*_ is the rate constant for the CETP dependent transfer of cholesterol to VLDL and *k*_*28*_ is the rate constant for the CETP mediated transfer of cholesterol to LDL. Finally, *k*_*29*_ is the rate constant for reverse cholesterol transport.

(22) $$\begin{array}{l} \frac{d\left[\text{HDLC}\right]}{\text{dt}}=k_{26}\left[\text{LCAT}\right]\left[\text{NHDL}\right]\left[PFC\right]\\ \;-k_{27}\left[CETP\right]\left[HDLC\right]\\ \;-k_{28}\left[CETP\right]\left[HDLC\right]\\ \;-k_{29}\left[HDLC\right]\left[SRB1\right]\;\left(\text{ODE}\;14\right) \end{array}$$

### Updating of ODEs 5 and ODE6

ODE 5 and ODE 6 were updated to include the changes described above. Again the system was re-parameterized and brought into a steady state to reflect these new changes. HDL-C reached a steady state which was based on the literature for normolipidemic male. All reactions and parameters are summarised in Table 2.

(23) $$\begin{array}{c} \frac{d\left[HFC\right]}{dt}=k_{19}+\left(k_{10}\left[HCE\right]\left[CEH\right]\right)-\left(\frac{BCR_{\max}}{1+{\left(BCR_{t}/HFC\right)}^{\mathrm{BS}}}\right)\\ \left(\frac{HCS_{\max}}{1+{\left(HFC/HCS_{t}\right)}^{\mathrm{HS}}}\right)+\left(k_{6}\left[IBS\right]\left[IC\right]\right)+\left(k_{16}\left[HLDLRs\right]\left[IDLC\right]\right)+\;\\ \left(k_{18}\left[HLDLRs\right]\left[LDLC\right]\right)+\left(k_{29}\left[HDLC\right]\left[SRB1\right]\right)\left(k_{14}\left[HLDLRs\right]\left[VLDLC\right]\right)\\ -k_{12}\left[HFC\right]-\left(\frac{k_{5}\left[HFC\right]}{\left[BSP\right]}\right)-k_{9}\left[ACAT\right]\left[HFC\right]\;\left(\text{Updated ODE}\;5\right)\\ \frac{d\left[PFC\right]}{dt}=k_{21}-k_{25}+k_{24}\left[PCE\right]\left[CEH\right]-k_{26}\left[LCAT\right]\left[NHDL\right]\left[PFC\right]-k_{23}\left[ACAT\right]\left[PFC\right]+\;\\ \frac{PCS_{\max}}{1+{\left(PFC/PFC_{t}\right)}^{\mathrm{PCSS}}}+k_{20}\left[LDLC\right][PLDLRs]\;\text{(Updated ODE}\;6) \end{array}$$

### Model exchangeability, SBML and submission to the Biomodels database

The model was coded into SBML using an export function in MathSBML (Additional file 2). Unlike computer languages such as C, C++ and Java, SBML was not designed to be coded manually; instead a number of tools are available that automatically generate SBML. The code can then be exchanged between tools supporting SBML [17]. In this case the model code was transferred into the software tool Copasi to facilitate parameter scanning [79]. Please note that when the ODEs are integrated in Copasi using the initial conditions, LDL-C enters a slightly higher steady-state than it does with MathSBML. In addition the model was submitted to the Biomodels database http://www.ebi.ac.uk/biomodels-main/ to facilitate its updating and future exchange (MODEL1206010000).

## Competing interests

The authors declared that they have no competing interests.

## Authors' contributions

MTM^c^A was responsible for the computational mathematical modeling and sensitivity analysis of the system. MTM^c^A was also responsible for data mining and acquisition of variable and parameter data. TBLK provided insight into the effects of ageing on the system, while DJW and JLJ advised on the modeling of the system. All authors read and approved the final manuscript.

## Supplementary Material

Additional file 1 Copasi file of Whole-Body Model of Cholesterol Metabolism. Click here for file

Additional file 2 SBML file of Whole-Body Model of Cholesterol Metabolism. Click here for file
